# Self-stigma, career development and employment in young adults with ASD in Chile.

**DOI:** 10.1192/j.eurpsy.2024.788

**Published:** 2024-08-27

**Authors:** M. S. Burrone, C. A. Barrientos, D. Saa-Ulloa, J. Madrid Lira, C. Alarcón, C. Cortés Rojas, L. D. Colantonio, M. T. Solís-Soto

**Affiliations:** ^1^Instituto de Ciencias de la Salud, Universidad de O’Higgins, Rancagua, Chile; ^2^University of Sydney, Sydney, Australia; ^3^University of Alabama at Birmingham, Birmingham, United States

## Abstract

**Introduction:**

Stigma describes prejudicial attitudes, negative stereotypes, and discrimination targeting a subgroup. Various forms of stigma have been identified in the literature, including self-stigma. Self-stigma or internalized stigma occurs when stigmatized individuals become aware of the negative stereotypes and apply these to themselves. Self-stigma may be a barrier to career development and employment in individuals with Autism Spectrum Disorder (ASD). However, there are few data available on the presence of self-stigma among young adults with ASD in Chile to inform local interventions and policies.

**Objectives:**

To analyze self-stigma and its relation with career development and employment in young adults with ASD in Chile.

**Methods:**

A mixed-method observational study was conducted to analyze self-stigma and its association with career development and employment among young adults with ASD in two regions of Chile. For the quantitative analysis, self-stigma was assessed using the Internalized Stigma of Mental Illness (ISMI) scale, and employment information was collected. For the qualitative analysis, in-depth interviews were conducted. Data from the interviews were digitalized and transcribed, and the analysis was conducted using ATLAs.ti following the principles of Glaser and Strauss’s Grounded Theory. All participants provided written informed consent, and the study was approved by the local Institutional Review Board.

**Results:**

Overall, 356 participants were included in the quantitative analysis (mean age: 27.8 [SD 6.2] years, 44.7% women, 14.8% with regular employment). The mean ISMI for the total sample was 2.34 (SD = 0.62). By triangulating this information with the qualitative analysis (n=27), it was observed that young adults with ASD frequently experience self-stigma attitudes. Through the in-depth interviews, we identified barriers and facilitators for the development of self-stigma. Also, we identified that negative self-perceptions among young adults with ASD may be a barrier to seeking career development opportunities and employment in this population.

**Conclusions:**

The current study shows self-stigma is present in young adults with ASD in Chile, and this may impact negatively their career development and employment.

**Disclosure of Interest:**

None Declared

